# Heterozygosity Maintains Developmental Stability of Sternopleural Bristles in *Drosophila subobscura* Interpopulation Hybrids

**DOI:** 10.1673/031.011.11301

**Published:** 2011-08-31

**Authors:** Zorana Kurbalija Novicic, Marina Stamenkovic-Radak, Cino Pertoldi, Mihailo Jelic, Marija Savic Veselinovic, Marko Andjelkovic

**Affiliations:** ^1^ Institute of Biological Research, University of Belgrade, Despot Stefan Blvd. 142, 11000 Belgrade, Serbia; ^2^ Faculty of Biology, University of Belgrade, Studentski trg 3, 11000 Belgrade, Serbia; ^3^ Department of Ecology and Genetics, Institute of Biological Science, University of Aarhus, Ny Munkegade, Building 540, DK8000 Aarhus C, Denmark; ^4^ Mammal Research Institute, Polish Academy of Sciences, 17-230 Białowieza, Poland

**Keywords:** developmental homeostasis, fluctuating asymmetry, genome coadaptation, hybridization, phenotypic variance

## Abstract

Interpopulation hybridization can lead to outbreeding depression within affected populations due to breakdown of coadapted gene complexes or heterosis in hybrid populations. One of the principal methods commonly used to estimate the level of developmental instability (DI) is fluctuating asymmetry (FA). We used three genetically differentiated *Drosophila subobscura* populations according to inversion polymorphism analysis and measured the variability of sternopleural bristle number and change in FA across generations P, F1, and F2 between intra- and interpopulation hybrids of *D. subobscura.* The mean variability of sternopleural bristle number in intra- and interpopulation hybrids of *D. subobscura* across generations cannot determine whether the changes at the level of developmental homeostasis are due exclusively to genomic coadaptation or to heterozygosity. Phenotypic variance (V_p_) and FA of sternopleural bristle number was higher in interpopulation than in intrapopulation hybrids across generations. F1 hybrids were more developmentally stable compared to each parental population in both intra- and interpopulation hybrids. The most probable mechanism providing developmental homeostasis is heterozygote or hybrid superiority, also called overdominace. However, V_p_ was higher and FA lower in the F2 generation when compared to F1, due mainly to crossing-over in the formation of F2.

## Introduction

Hybrid zones occur when two genetically distinct populations meet, mate, and produce offspring of mixed ancestry (Barton and Hewitt 1985; Ross and Robertson 1990). These zones can occur in disrupted habitats through interpopulation hybridization, where previously isolated populations become intermixed and hybridize. Anthropogenic activities in natural ecosystems may increase opportunities for interpopulation hybridization through habitat disruption. Hybridization between individuals from different populations can lead to heterosis, also called overdominace, in the first generation, followed by outbreeding depression in the consecutive generation due to the breakdown of coadapted gene complexes (Dobzhansky 1950; Andersen et al. 2002; Edmands 2007). There is growing evidence that environmental and genomic stressors may induce significant levels of developmental instability (DI) (Palmer and Strobeck 1986; Palmer 1996; Møller and Swaddle 1997; Pertoldi et al. 2006a; Kurbalija et al. 2010), suggesting that DI could serve as an early signal in monitoring the effects of stress in populations (Leary and Allendorf 1989).

Developmental stability is the ability of an organism to buffer environmental and genetic disturbances which affect the developmental capacity of a particular phenotype (Zakharov 1981; Palmer 1996). The failure to correct for random accidents during development may be manifested as fluctuating asymmetry (FA), which is defined by small deviations from perfect bilateral symmetry of morphological structures (Leary and Allendorf 1989; Palmer and Strobeck 1986).

The genetic basis of developmental stability has been much debated over the last five decades. The increase or decrease of DI as a consequence of the genomic stress has been explained by two major hypotheses. The first argues that the level of stability is a reflection of the underlying genomic heterozygosity (Lerner 1954), while the second argues that stability reflects the general level of genomic coadaptation (Dobzhansky 1950).

The coadaptive gene complexes are established over the evolutionary history of the genome via natural selection and are defined as a specific balance between loci within the genome (Dobzhansky 1950). Such coordination within the genome protects individuals from developmental accidents, which can be caused by both environmental and genetic factors (Parsons 1990). A breakdown of coadaptation can be manifested in an individual as a decreased ability to develop an optimal phenotype due to increased DI (Leary et al. 1985; Markow 1995).

Heterozygosity theory predicts that the level of heterozygosity is correlated with DI (Lerner 1954; Livshits and Kobyliansky 1985), such that heterozygous individuals are better adapted to genomic and environmental perturbations than homozygous individuals due to higher developmental stability. The theory also states that single loci coding for enzymes related to metabolic efficiency influence developmental stability of different morphometric characters (Palmer 1996; Møller and Swadle 1997), predicting that levels of heterozygosity at loci coding for functional proteins will be inversely correlated with level of DI (Lerner 1954; Livshits and Kobyliansky 1985). However, an important assumption is that heterozygosity of various genetic markers accurately reflects the heterozygosity of the entire genome, or at least the heterozygosity of the loci that contribute to the formation of the morphological phenotype (Mitton and Grant 1984; Chakraborty 1987).

Whether heterozygosity and/or genomic coadaptation influence parameters of developmental stability is still unclear (Pertoldi et al. 2006b). Available data indicate a tendency of FA to increase with inbreeding (Palmer and Strobeck 1986; Waldmann 1999, 2001; Babbitt 2006; Fessehaye et al. 2007), hybridization between species (Hochwender and Fritz 1999; Stamenkovic-Radak et al. 2009), or between populations (Waldmann 1999; Kurbalija et al. 2010), although several studies report exceptions to this pattern (Clarke et al. 1992; Sheridan and Pomiankowski 1997; Trotta et al. 2005). A consensus does appear to exist in evidence that any stress would increase phenotypic variance (V_p_) of most quantitative traits (Bijlsma and Loeschcke 1997; Hoffmann and Parsons 1997; Hoffmann and Hercus 2000; Pertoldi et al. 2001a,b).

It is well known among *Drosophila* spp. that Vp of individuals collected in the field is greater than those reared in a laboratory setting (Coyne and Beecham 1987; Imasheva et al. 1994; David et al. 1997; Gibert et al. 1998; Gibert et al. 2004). The higher phenotypic variability in flies from the wild is generally attributed to higher environmental variance, resulting in lower heritability (Coyne and Beecham 1987; Gibert et al. 1998; Hoffmann and Hercus 2000). It has been suggested that several mechanisms can alter the components of V_p_ (Waddington 1942), and recent studies have aimed to identify their respective roles within an evolutionary context (e.g. Zakharov 1992; Debat and David 2001; Meiklejohn and Hartl 2002).

Sternopleural bristle number is a widely studied characteristic in quantitative genetics (Mackay 2004) that displays a rapid response to artificial selection in *Drosophila* spp. (Ramuson 1955; Barnes and Kearsey 1970; Schnee and Thompson 1984). The genetic variance of SB obtained from IF analysis in *Drosophila melanogaster* was higher at extreme temperatures (Imasheva et al. 1998; Pétavy et al. 2006). Some studies show a positive correlation between FA of sternopleural bristle number and temperature variations (Parsons 1961; Imasheva et al. 1997; Bubliy et al. 2000; Pétavy et al. 2006) and nutritional variations as stress conditions (Imasheva et al. 1999). The results of these studies support the rather popular point of view that environmental stress is associated with higher FA levels (Møller and Swaddle 1997). On the contrary, Woods et al. (1999) reported that stress did not induce an increase in the V_p_ or FA of sternopleural bristle number in *D. melanogaster.* Despite heterogeneity among results in the association between different kinds of environmental stresses and FA and V_p_ as estimators of DI, evidence is scarce regarding the effects of interspecies or interpopulation hybridization as a genomic stressor and its correlation with FA of this meristic trait in *Drosophila* species (Gilligan et al. 2000; Andersen et al. 2002; Vishalakshi and Singh 2008).

Inversion polymorphism of different *Drosophila* species was used as a model system for studying processes involved in adaptation and genetic diversity (Balanya et al. 2004; Hoffmann et al. 2004; Stamenkovic-Radak et al. 2008). As crossing-over is suppressed within the inversion loops of heterokaryotypes, all genes within the inverted segments segregate as a linked group representing one physical and functional unit, called the supergene, the different arrangements of which can be referred to as allelic complexes (Krimbas 1993; Rasic et al. 2008; Santos 2009). *D. subobscura* is a Palearctic species that displays rich inversion polymorphism in all five acrocentric chromosomes of the set (Krimbas and Loukas 1980; Krimbas 1992, 1993). Assuming a relatively long-time of selection on the linked genes within inverted regions, *D. subobscura* represents a suitable species for testing the heteozygosity vs. coadaptation hypotheses.

This paper focuses on the coadaptive aspect of inversion polymorphism in *D. subobscura* populations from three ecologically and topologically distinct habitats, which possess a certain degree of genetic differentiation due to their different evolutionary histories. The aim of the study is to investigate the level of DI estimated by FA and V_p_ in sternopleural bristle (SB) number in intra- and interpopulation hybrids through 3 generations (P, F1 and F2) in isofemale lines (IF) of *D. subobsura.* The study intends to contribute to understanding the effects of coadaptations and heterozygosity on the level of developmental homeostasis in intra- and interpopulation hybrids.

## Materials and Methods

### Population samples

For the present study, *D. subobscura* individuals were sampled in Serbia using fermented fruit traps. The flies were collected from three localities (beech-B, oak-O and Botanical Garden-BG). The first locality, Beech wood (B) (*Abieto-fagetum*), is situated between N 43°33′ 28.43″ and E 20°45′ 10.96″ (Mountain Goc, Central Serbia). The second is Oak wood (O) (*Fraxineto-quercetum*) situated between N 43°32′ 57.38″ and E 20°40′ 2.32″ (Mountain Goc, Central Serbia).

These two woods have distinctive microclimates; Beech wood has the highest altitude (875 m) and the highest humidity with great vegetation coverage, while Oak wood (787 m) has more sparsely distributed trees and is slightly warmer. The third locality is the Botanical Garden at the University of Belgrade (BG) (*Arboretum-Corilus colurna, Celtis australis*), situated between N 44° 49′ and E 20 ° 28′ at 87 m above sea level, representing a more urbanized environment with a unique microclimate and high anthropogenic influence.

The females collected at these three localities were individually used to obtain isofemale lines (IF) reared on the common cornmeal-sugar-yeast-agar medium for *Drosophila.* The progeny of these IF lines were used as the parental generation in the experiment in order to preserve the high amount of genetic variability from natural population. All cultures were maintained and all experiments performed under constant laboratory conditions at 19°C, approximately 60% relative humidity, light of 300 lux and 12:12 L:D cycles.

### Population genetic structure and data analysis

Analysis of inversion polymorphism was carried out for the wild captured *D. subobscura* males, which were individually crossed with virgin females from Küsnacht laboratory stock, homozygous for Standard gene arrangement at all five large chromosomes. Salivary glands from third-instar larvae were squashed and chromosomes stained with aceto-orcein solution. Eight larvae were analyzed from the progeny of each of the crosses performed. The chromosome map from Kunze-Muhl and Muller (1958) was used for the cytological analysis of gene arrangements. The designation of gene arrangements followed that of Kunze-Muhl and Sperlich (1955). The analysis included in total 56 males (112 autosomes, 56 sex chromosomes) from Oak population, 44 males from Beech population (88 autosomes, 44 sex chromosomes), and 52 males from Botanical Garden (104 autosomes, 52 sex chromosomes).

Z-statistics (Zar 1999) were used to assess the differences between frequencies of gene arrangements in the pairs of analyzed populations. The G-test (Sokal and Rohlf 1980) was used to determine population subdivision by determining the homogeneity of gene arrangement frequencies between pairs of populations from different forest communities on all chromosomes and autosomes.

### Experimental design

The experiment used 63 IF lines from Oak, 38 from Beech, and 64 from the Botanical Garden; the progeny of these IF lines were used as the parental (P) generation in the experiment. Virgin males and females were separated within each IF line upon emerging and intra- and interpopulation crosses were made four days after eclosion.

Intrapopulation (B × B, O × O, BG × BG) and interpopulation crosses (B × O, BG × O, BG × B) were made among IF lines of the three *D. subobscura* populations. Both direct and reciprocal crosses were made in order to take into account any maternal effect (Table 1). The progeny (six males, six females) from each cross were transferred to fresh vials to obtain F1 and F2 generations. Individuals in P, F1, and F2 generations from intra- and interpopulation crosses (B × B, O × O, BG × BG, B × O, BG × O, BG × B) were frozen at -20°C and used for further sternopleural bristles counting procedure.

### Count of sternopleural bristle number

The left and right side of the sternopleural region from each fly was observed under a 100× binocular microscope and the SB number was scored and counted twice, first on the right side (R), then on the left side (L). This meristic characteristics and others may be measured without error thus avoiding the need for replicate measurements (Palmer 1994). According to most other similar investigations, all short, medium-sized, and long bristles were counted.

### Fluctuating asymmetry data analyses

Before interpreting FA population estimates, several statistical assumptions were made. In the folowing statistical analysis, males and females from each population, each generation, and direct and reciprocal crosses were analyzed separately. Because of the large number of tests conducted, the sequential Bonferroni test (Rice 1989) was used. Fluctuating asymmetry is characterized by normal distribution of the right-side minus left-side differences with a mean of zero and a normal distribution (Palmer and Strobeck 1986). No deviations from normal distribution was found using Shapiro-Wilk (W) and Chisquared (χ^2^) tests.. One-sample *t*-tests were performed to test for a departure from the mean of (R — L) from the expected mean of zero; no directional asymmetry was detected in all tested samples.Tests for correlation between SB number and FA were done because significant correlation may affect results and interpretation (Palmer 1994; Palmer and Strobeck 2003). Linear dependence of FA on the mean SB number was tested by linear regression; FA acted as the explanatory variable and SB number as the dependent variable. In 43.05% cases we found significant positive corelation between FA and SB number (Table 2).

Because higher variance was typically observed in cases where more bristles were found, a log transformation was used on variance values and further tests were done with transformed values to account for a scaling effect of the mean. The FA1 index (Palmer 1994) was measured as |R — L| between sides in all samples of intrapopulation and interpopulation hybrids, both direct and reciprocal crosses, separately for males and females through P, F1, and F2 generations. The FA1 index is the one of the most frequently used indices for describing the level of FA in a sample. It also acts as an unbiased estimator of the sample standard deviation, and is recommended for testing FA differences between three or more samples (Palmer and Strobeck 1992). However, because of the significant presence of positive correlation between FA and SB number, we also used FA2 index (Palmer 1994). The FA2 index (mean (|R — L|/((R+L)/2))) is commonly used only where clear evidence exists of a size dependence of |R — L| among individuals within a sample.

The multivariate ANCOVA was conducted to determine effects of intra- and interpopulation hybridization, generation, sex as an independent variable, and direct and reciprocal crosses as covariate variables while controlling for FA1, FA2, and mean sternopleural bristle number, the latter acting as a dependent variable. The analysis was performed using general multivariate linear model in SPSS software.

Student *t*-tests and *F*-tests were used to test differences in mean and variances of the sternopleural bristle number and FA between sexes, populations, generations, and type of cross. All tests treated sex, population, generation, and type of cross as separate variables. The conservative *F*-test and *t*-test were used to reduce the possibility of a Type 1 error. Furthermore, we have performed corrections for multiple comparisons and therefore our results are both conservative and robust, and not affected by eventual interactions between the different factors. All statistical analyses were performed using PAST software (Hammer et al. 2001).

## Results

### Population genetic structure

Frequencies of gene arrangements and parameters of inversion polymorphism (Degree of Heterozygosity, Inversion Density, Index of Free Recombination) on five acrocentric chromosomes of *D. subobscura* population from three different natural habitats are shown in Table 3. Inversion polymorphism analysis of Beech population (B) detected 15 stuctural chromosomal types and 13 inversions; in the Oak (O) and the Botanical garden populations (BG), 16 stuctural chromosomal types and 15 inversions were detected.

Results of the *G*-test gave the level of interpopulation differences in gene arrangement distribution for each individual chromosome. The results showed significant differences in gene arrangement distribution between B and O populations for four chromosomes: A (G = 10.91, *p* < 0.01), J (G = 14.49, *p* < 0.01), U (G = 11.10, *p* < 0.01), and E (G = 9.52, *p* < 0.05), and for all chromosomes in total (G = 49.24, *p* < 0.01) (Table 4).

Interpopulation differences in gene arrangement distributions of each chromosome were found between B and BG populations: U (G = 9.70, *p* < 0.01), E (G = 12.84, *p* < 0.01), O (G = 14.44, *p* < 0.01), and for all chromosomes in total (G = 39.25, *p* < 0.01) (Table 4).

Interpopulation differences in gene arrangement distributions on each chromosome were found between O and BG populations: J (G = 17.02, *p* < 0.01), U (G = 30.44, *p* < 0.01), E (G = 31.23, *p* < 0.01), O (G = 24.53, *p* < 0.01), and for all chromosomes in total (G = 116.95, *p* < 0.01) (Table 4).

The results of the *Z*-test showed the level of interpopulation differences in individual gene arrangement frequencies and revealed significant differences in some gene arrangement frequencies between each pair of tested populations (B and O, B and BG, O and BG) (Table 5).

The results showed significant differences in some gene arrangement frequencies for all chromosomes of the set between some populations (B and O, BG and O). There were significant differences in gene arrangement frequencies between B and BG populations, for U, E and O chromosomes (Table 5). Gene arrangement E_1+2+9+12_ was detected only in O population, as well as gene arrangement O_6_ which was detected only in BG population with a frequency of less than 3%.

### Multivariate testing of fluctuating asymmetry and mean of SB number

The results of the multivariate test using a general linear model are presented in Table 6. Significant differences were detected between populations; intrapopulation and interpopulation hybrids (F = 29.524, *p* < 0.01), generations (F = 113.114, *p* < 0.01), and type of cross (F = 2.995, *p* < 0.05). Significant interactions were also found between population × generation (F = 59.919, *p* < 0.01) and generation × sex (F = 11.022, *p* < 0.01).

The results test of between-subject effect are shown in Table 7. The significant differences were found between populations (FA1, F = 22.471, *p* < 0.01; FA2, F = 20.153, *p* < 0.01; mean SB number, F = 19.895, *p* < 0.01), generations (FA1, F = 49.513, *p* < 0.01; FA2, F = 85.854, *p* < 0.01; mean SB number, F = 65.351, *p* < 0.01). Significant interaction was also found between population × generation (FA1, F = 15.901, *p* < 0.01; FA2, F = 13.197, *p* < 0.01; mean SB number, F = 44.140, *p* < 0.01).

### Univariate testing of fluctuating asymmetry and mean of SB number Intrapopulation hybridization.

***Changes of mean and variance across generations in males.*** Analysis of the difference in mean and variance of the average SB number in males is given in Table 8a. Generally, very low variability in sternopleural bristle number in *D. subobscura* populations was observed. A significant increase of the average SB number was detected through generations (P < F1 < F2) in O × O hybrids resulting from direct cross. In B × B hybrids, a significant increase in SB number was detected in the F1 generation when compared to the parental generation, followed by a decrease in SB number in the F2 generation in both direct and reciprocal crosses.

In BG × BG hybrids, a trend of constant increase in SB number was detected across generations (P < F1 < F2) in both direct and reciprocal crosses.

No significant change in variance across generations was detected except in the case of BG × BG hybrids for both direct (F_P,F1_ = 1.58, *p* < 0.05; F_F1,F2_ = 1.45, *p* < 0.01) and reciprocal crosses (F_P,F1_ = 1.69, *p* < 0.05; F_F1,F2_ = 1.32, *p* < 0.05).

***Changes of mean and variance across generations in females.*** Analysis of the difference in mean and variance of the average SB number in females is given in Table 8b. The statistically significant increase of SB number was successive through generations for all hybrid groups in both direct and reciprocal crosses. Regarding the variance, no statistically significant change was detected across generations in all tested groups of intrapopulation hybrids for females.

***Changes of FA across generations in males.*** Analysis of the FA1 index between generations for SB number in males shows no significant difference between generations in the direct cross. There was, however, significant decrease of FA value across generations in O × O (t _P,F1_ = 3.48, *p* < 0.01; t _P,F2_ = 4.00, *p* < 0.01) and B × B (t _F1,F2_ = 2.41, *p* < 0.05) hybrids in reciprocal crosses (Table 9a).

Analysis of the FA2 index between generations for SB number in males shows no significant difference between generations in the direct cross, although there was significant decrease of FA value across generations in O × O (t _P,F1_ = 2.84, *p* < 0.01; t _P,F2_ = 3.66, *p* < 0.01) hybrids in reciprocal crosses (Table 8a).

***Changes of FA across generations in females.*** Analysis of FA differences between generations using FA1 index for SB number in females shows no significant difference between generations in both direct and reciprocal crosses except in the case of B × B hybrids (t _P,F1_ = 2.03, *p* < 0.05) in direct cross and O × O hybrids (t _F1,F2_ = 3.38, *p* < 0.01) in reciprocal cross (Table 9b).

Analysis of FA differences between generations using FA2 index for SB number in females shows no significant difference between generations in both direct and reciprocal crosses, except in the case of O × O hybrids in reciprocal cross, where FA value significantly decreased across generations (t _F1,F2_ = 3.69, *p* < 0.01). In the case of B × B hybrids, a constant decrease of FA across generations was detected (t _P,F2_ = 2.60, *p* < 0.05; t _F1,F2_ = 2.41, *p* < 0.05) (Table 10b).

### Inter-population hybridization

***Changes of mean and variance across generations in males.*** Analysis of the difference in mean and variance of the average SB number in males is given in Table 8a. The results showed significant differences in SB number and variance in most interpopulation hybrids groups in both direct and reciprocal crosses. In B × O hybrids an increase in SB number in F1 generation was detected when compared to the parental generation, followed by a significant decrease in the F2 generation (t _F1,F2_ = 2.35, *p* < 0.05) in direct crosses. A different trend was found for reciprocal crosses (P > F1 > F2). In the case of BG × O hybrids, the significant increase in average SB number across generations was detected (P < F1 < F2) in direct crosses. In BG × B hybrids, a significant decrease of SB number in the F1 generation was found when compared to the parental generation, followed by a significant increase in SB number in the F2 generation for both direct and reciprocal crosses.

The variance in SB number in B × O hybrids showed a statistically significant increase in F2 compared to F1 generation in both direct (F _F1,F2_ = 1.83, *p* < 0.01) and reciprocal crosses (F _F1,F2_ = 1.16, *p* < 0.01). In the case of BG × O hybrids, the variance showed a significant constant increase across generations (P < F1 < F2) for both direct and reciprocal crosses. In the BG × B hybrids a significant increase of variance was detected in F2 when compared to F1 generation in reciprocal crosses (F _P,F2_ = 1.82, *p* < 0.05; F _F1,F2_ = 1.61, *p* < 0.01).

***Changes of mean and variance across generations in females.*** Analysis of the difference in mean and variance of the average SB number in females is given in Table 8b. In B × O hybrids an increase of average SB number was detected in the F1 generation when compared to the parental generation followed by a decrease in the F2 generation in reciprocal crosses. In BG × O hybrids a statistically significant increase of SB number was detected across generations (P < F1 < F2) in both direct and reciprocal crosses. In BG × B hybrids a significant decrease in average SB number was detected in the F1 generation when compared to the parental generation, and an increase in the same value for the F2 generation (P > F1 < F2) in both direct and reciprocal crosses

A decrease in variance was detected in the F1 generation followed by a significant increase in the F2 generation for B × O hybrids in both direct (F _P,F2_ = 1.71, *p* < 0.05; F _F1,F2_ = 2.03, *p* < 0.01) and reciprocal crosses (F _P,F2_ = 2.73, *p* < 0.01; F _F1,F2_ = 2.93, *p* < 0.01). In BG × O and BG × B hybrids a significant increase of variance was detected in the F2 generation when compared to the F1 generation in both direct and reciprocal crosses.

***Changes of FA across generations in males.*** Analyses of the FA1 index between generations for sternopleural bristles in males are given in Table 9a. The results showed a statistically significant decrease in FA values across generations in B × O hybrids in both direct (t _P,F2_ = 4.50, *p* < 0.01; t _F1,F2_ = 6.03, *p* < 0.01) and reciprocal crosses (t _P,F2_ = 4.80, *p* < 0.01; t _F1,F2_ = 7.07, *p* < 0.01). In BG × O hybrids a significant decrease of FA was detected in the F2 generation when compared to the F1 generation in both direct (t _F1,F2_ = 2.19, *p* < 0.05) and reciprocal crosses (t _F1,F2_ = 2.86, *p* < 0.01). In BG × B hybrids a significant increase of FA was detected across generations in both direct (t _P,F2_ = 2.57, *p* < 0.05) and reciprocal crosses (t _P,F1_ = 2.44, *p* < 0.05).

Analyses of the FA2 index between generations for SB number in males are given in Table 10a. The results showed a statistically significant decrease of FA values across generations in B × O hybrids in both direct (t _P,F2_ = 5.41, *p* < 0.01; t _F1,F2_ = 6.44, *p* < 0.01) and reciprocal crosses (t _P,F2_ = 4.67, *p* < 0.01; t _F1,F2_ = 7.50, *p* < 0.01). In BG × O hybrids a decrease of FA values was detected in the F2 generation when compared to the F1 generation in reciprocal crosses (t _F1,F2_ = 3.17, *p* < 0.05). In BG × B hybrids a decrease of FA values was detected across generations in reciprocal crosses (t _P,F1_ = 2.22, *p* < 0.05; t _P,F2_ = 2.60, *p* < 0.01).

***Changes of FA across generations in females.*** The results of FA1 analyses between generations for SB number in females are given in Table 9b. The results showed a significant increase in FA for B × O hybrids in the F2 generation when compared to the F1 generation in both direct (t _F1,F2_ = 7.16, *p* < 0.01) and reciprocal crosses (t _F1,F2_ = 6.59, *p* < 0.01). In BG × O hybrids a significant decrease of FA was found in reciprocal crosses (t _F1,F2_ = 3.19, *p* < 0.01). In BG × B hybrids a significant decrease of FA was found across generations in direct crosses (t _P,F2_ = 2.57, *p* < 0.05).

The results of FA2 analyses between generations for SB number in females are given in Table 10b. The results showed an increase in FA value in the F1 generation when compared to the parental generation, along with a significant decrease in the F2 generation for B × O hybrids (P < F1 > F2) in both direct and reciprocal crosses. In BG × O hybrids a significant decrease of FA was found in the F2 generation when compared to the F1 generation in direct crosses (t _F1,F2_ = 3.08, *p* < 0.01), while a statistically significant decrease in FA across generations was found in reciprocal crosses (t _F1,F2_ = 2.63, *p* < 0.01;t _F1,F2_ = 3.68, *p* < 0.01). In BG × B hybrids a statistically significant decrease of FA was detected across generations in direct crosses (t _P,F2_ = 3.10, *p* < 0.01; t _F1,F2_ = 3.58, *p* < 0.01). The same trend of FA variability across generations was also found in reciprocal crosses (t _F1,F2_ = 1.97, *p* < 0.001).

## Discussion

The results showed that populations of *D. subobscura* from three ecologically and topologically distinct habitats possess a certain degree of genetic difference, likely due to their different evolutionary histories. The gene arrangements are carriers of various alleles that are differently favored in diverse environmental conditions and prove in most cases to be the major factor determining the gene arrangement frequencies in natural populations of *D. subobscura* (Andjelkovic et al. 2003). The results presented here further emphasize the relationship between gene arrangement frequencies and local adaptation to ecologically different microhabitats in *D. subobscura.*

The inversion polymorphism analysis showed that analysed populations differ in the frequencies of some gene arrangements that are in agreement with previous results (Andjelkovic et al. 2003; Stamenkovic-Radak et al. 2008; Jelic et al. 2009). In general, the pattern of inversion polymorphism of the three analyzed *D. subobscura* populations was consistent with the observed inversion polymorphism of *D. subobscura* in the southeast margin of the Central European region (Krimbas 1993). Therefore, the results suggest that the three populations in this study are suitable for testing the coadaptation vs. heterozygosity hypothesis.

This paper focuses on the coadaptive aspect of genetic variability at the population level and its relationship to interpopulation hybridization as a genomic stressor. The trait used in the study was a typical meristic character widely considered as a threshold trait (Falconer and Mackay 1996; Mackay 2001, 2004). Some data suggest the existence of particular genes that affect the genotype × environmental covariance of SB number (Kristensen et al. 2005; Sørensen et al. 2007). The underlying continuous variable is both genetic and environmental in origin and could be measured and studied equally as a metric character but with greater precision because of non-existence of measurement error.

The results regarding the mean of SB number for intra- and interpopulation hybrids of *D. subobscura* cannot explicitly reveal the significance of either of the two hypotheses, since no general trend has emerged for either intra- and interpopulation hybrids. However, the observed variance of the SB number across generations gives a completely different insight.

The variance of SB number after intra- and interpopulation hybridization showed that intrapopulation hybrids have no significant change of variance across generations in most of the hybrid groups, compared to interpopulation hybrids. However, a significant change of variance across generations is observed in interpopulation hybrids. A reduction of V_p_ in the F1 generation compared to the parental generation was obtained in most of the crosses, signifying that the hybrids had reached a higher level of homeostasis in the F1 generation, likely due to higher heterozygosity. Also, a significantly higher level of V_p_ in the F2 generation was obtained when compared to the F1 generation, probably due to disruption of coadapted gene complexes, which can be attributed to crossing over in formation of most of the genotypes in the F2 generation.

The analysis of the variability of FA of SB number using FA1 and FA2 indices (Palmer 1994) after intra- and interpopulation hybridizations showed that intrapopulations hybrids are relatively stable. Analysis showed no significant differences in FA in most of the cases between generations when compared to the interpopulation hybrids across generations. This result was expected when the effects of intrapopulation crosses are taken into account, which simulate random mating in natural populations. These crosses represent control conditions for genomic stress. On the other hand a significant trend of change in FA was observed across generations in interpopulation hybrids, which was not observed in intrapopulation hybrids.

Theoretically, a heterosis in the F1 generation and outbreeding depression caused by disruption of coadaptive gene complexes in the F2 generation for interpopulation hybrids was expected, but results showed a post-hybridization decrease in FA for interpopulation hybrids in both F1 and F2 generations.

The results showed a significant decrease of FA through generations each with the lowest FA detected in the F2 generation in all interpopulation hybrids. Therefore, it is likely that the observed difference between intra- and interpopulation hybrids regarding level of FA variability is due to increased heterozygosity in the F1 generation in interpopulation hybrids, and thus the increased developmental homeostasis and reduced FA. An increase of heterozygosity also reduced the FA in the F2 generation, which could be a result of selection pressure acting on F2 genotypes. Selection could eliminate developmentally unstable and extreme phenotypes from the population at very early stage and overall hybrid fitness could increase (Polak et al. 2002). The increase of FA in the F2 generation compared to the F1 generation was not obtained, indicating the possibility that disruption of coadaptation occurred and the less stable genotypes produced by crossing over were selected against. Therefore, in the F2 generation, we obtained only individuals with suitable gene combinations and higher developmental stability. This suggests that the most probable mechanism providing developmental homeostasis in a population is the heterozygote or hybride superiority, also known as overdominance.

Heterosis can be considered the opposite of inbreeding depression; both mechanisms share the same underlying causes (Crow 1993). The primary cause of heterosis is the sheltering of deleterious recessive alleles in hybrids. In addition, increased heterozygosity would increase the fitness of hybrid individuals at loci where heterozygotes have a selective advantage over homozygote types. A subdivision of natural populations can provide an appropriate condition for heterosis, as different deleterious recessive alleles could drift to relatevely high frequencies in different subpopulations. Therefore interpopulation hybrids produced by mating between immigrant individuals are expected to have greater fitness than residents (Whitlock et al. 2000; Morgan 2002).

The evidence for heterosis without coadaptation in *Drosophila ananassae* was presented in Sing (1972, 1985), showing that interracial hybridization does not lead to breakdown of heterosis associated with cosmopolitan inversions. These results provide observations that may carry weight on testing heterozygosity vs. coadaptation, but the findings of heterosis in interpopulation hybrids does not exclude hybrid breakdown in subsequent generations (Lynch and Walsh 1997).

In response to genomic stress, no significant difference was found between the majority of direct and reciprocal crosses, suggesting that parental population origin had no significance and that both sexes equally contribute to the inheritance of SB number. This suggests that sternopleural bristles, as a bilaterally symmetrical morphological trait, might be a good and consistent characteristic for measuring the level of developmental stability under genomic stress without a confounding maternal effect. These results contrast with wing FA in a previous study (Kurbalija et al. 2010).

There may be several explanations for the observed difference in the response of different bilateral symmetrical traits to genomic stress conditions. The development of distinct traits is probably under control of different gene complexes (Parsons 1990). A stress factor may also be specific for particular metabolic pathways and may not affect the FA of all traits in the same way (Palmer and Strobeck 1986). Furthermore, different traits are under different natural selection pressure that depends on functional importance of a given trait. Sternopleural bristles are not generally regarded as a fitness-related trait and we do not expect them to be a highly canalized trait.

Despite the fact that different outcomes can be expected after interpopulation hybridization, depending on evolutionary histories of populations and trait specific response, our results confirmed that DI could be a useful indicator of genomic stress in populations. Further knowledge on the consequences of interpopulation hybridization between wild populations as well as the capacity of small populations to adapt to local environmental conditions is urgently needed.

The integration of experimental, theoretical, and applied conservation genetics will contribute to understanding of the methodological and applied aspects of conservation genetics. It seems that *Drosophila* is a useful species to bridge the gap between theoretical computer simulation and studies on natural populations.

**Table 1.**
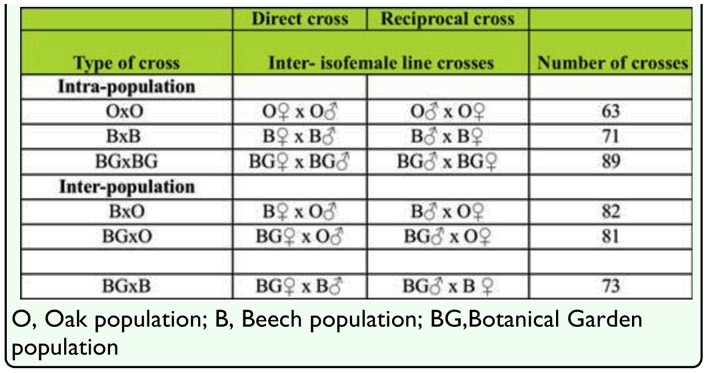
Number and type of intra- and inter-population crosses for direct and reciprocal crosses.

**Table 2.**
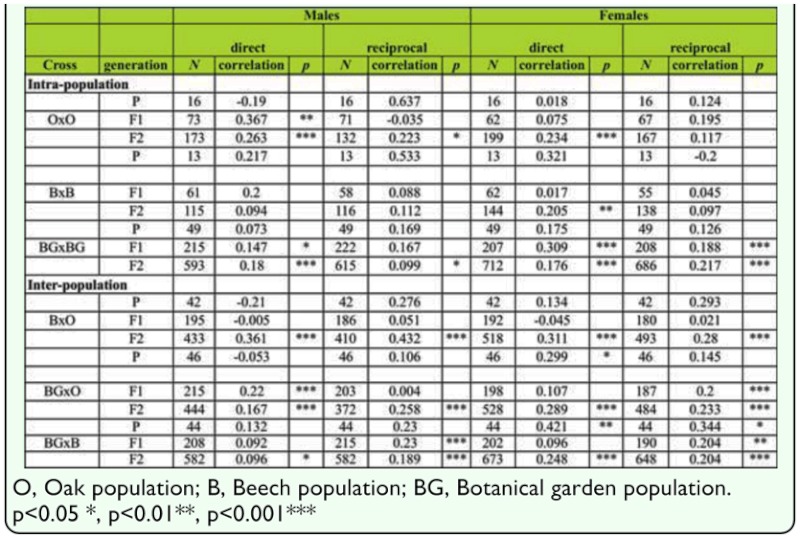
Tests for linear dependence of FA on SB average number using linear regression, of intra- and inter-population crosses in P, F1 and F2 generations in males and females (direct and reciprocal crosses).

**Table 3.**
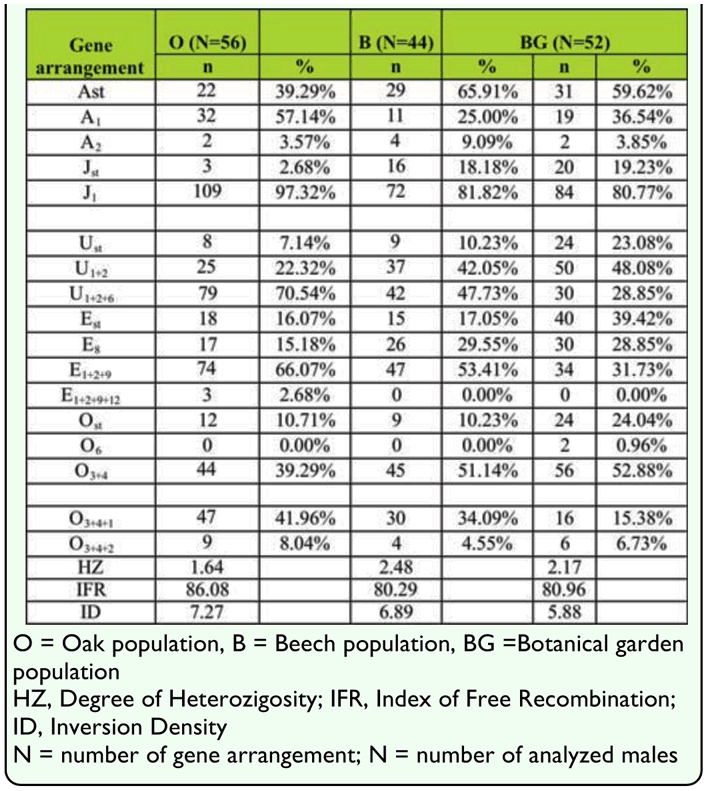
Gene arrangement frequency (%) and inversion polymorphism parameters of *Drosophila subobscura* populations from three different habitats.

**Table 4.**
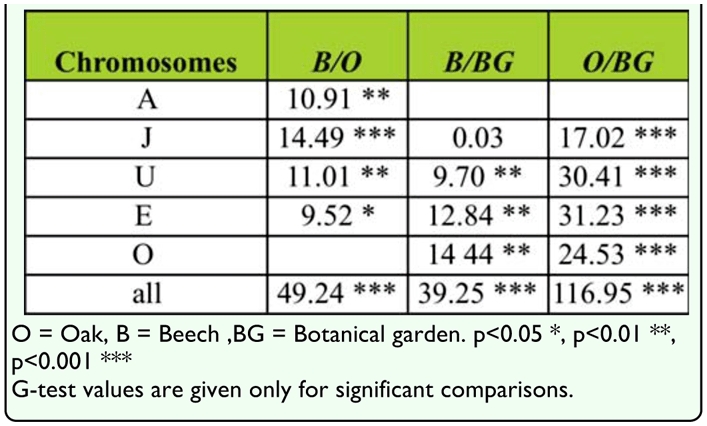
Chromosomal differences in arrangement frequencies between three analyzed habitats of *Drosophila subobscura* populations.

**Table 5.**
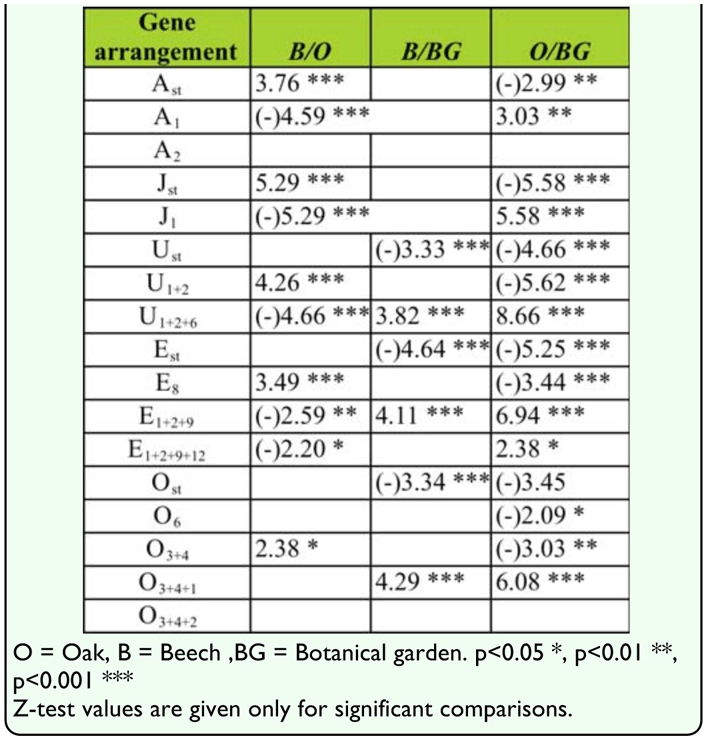
Differences in gene arrangement frequencies between three analyzed habitats of *Drosophila subobscura* populations.

**Table 6.**
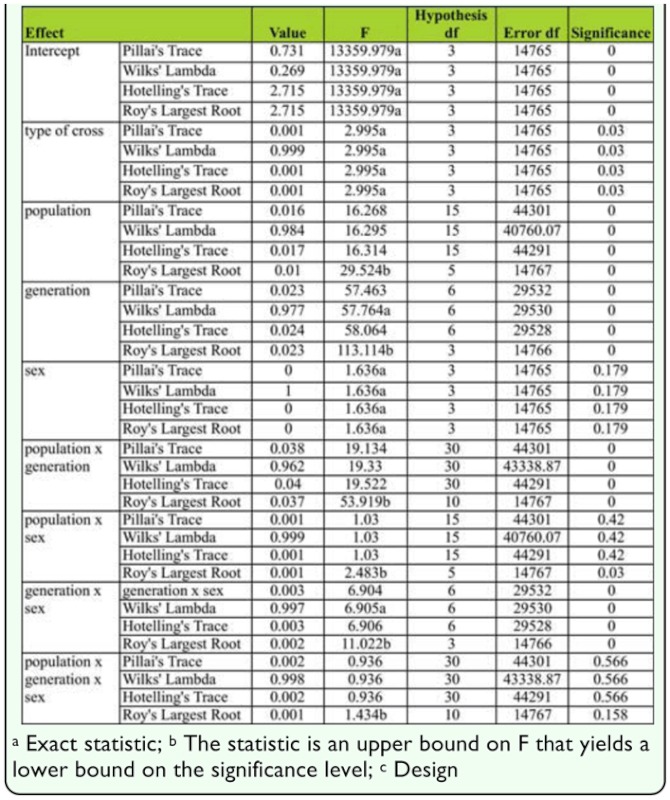
Results of multivariate test using General linear model.

**Table 7.**
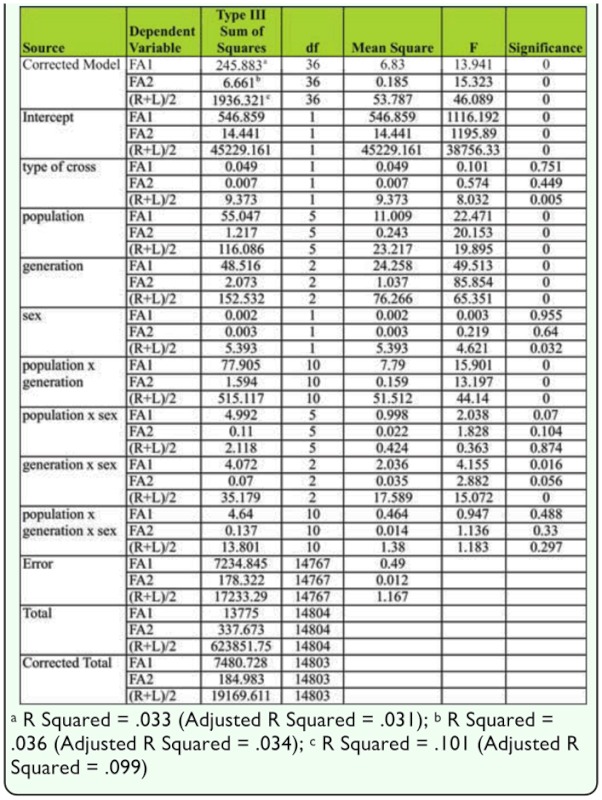
Results of tests of between-subjects effects using genelar linear model (multivariate).

**Table 8a.**
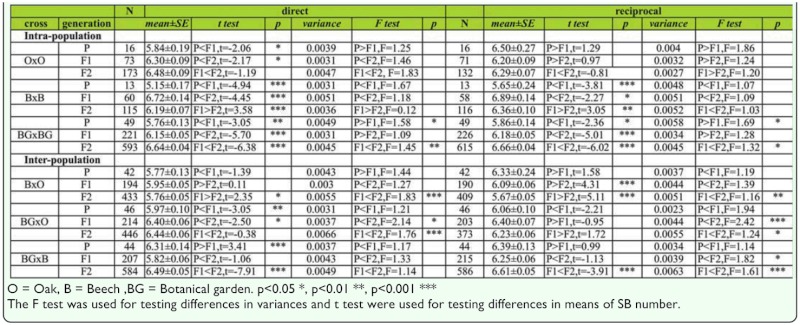
The mean and variance (log transformed) for sternopleural bristle number in males of intra- and interpopulation crosses across generations (direct and reciprocal crosses).

**Table 8b.**
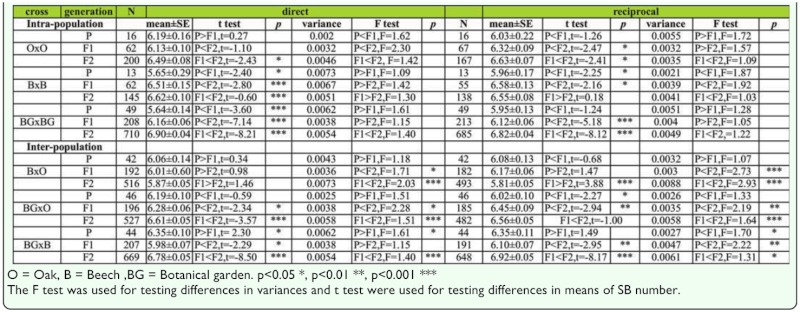
The mean and variance (log transformed) for sternopleural bristle number in females of intra- and interpopulation crosses across generations (direct and reciprocal crosses).

**Table 9a.**
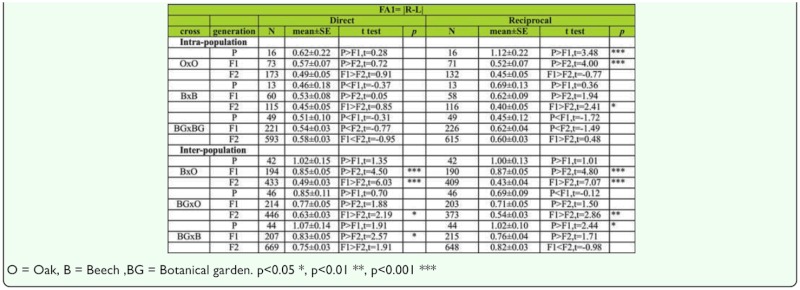
The FA1 index differences between generations and type of crosses for sternopleural bristles in males (direct and reciprocal crosses); *t test* were used for testing differences in mean of SB.

**Table 9b.**
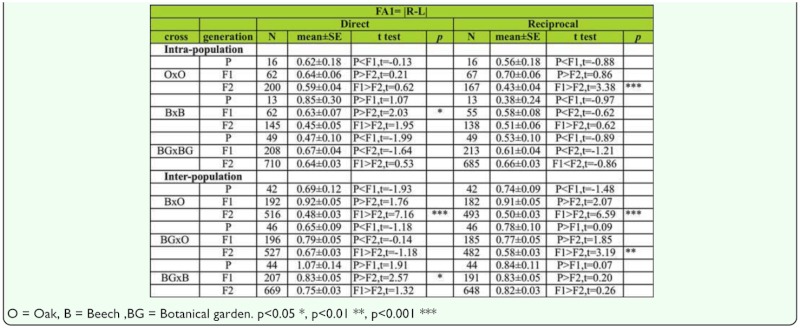
The FA1 index differences between generations and type of crosses for sternopleural bristles in females (direct and reciprocal crosses); *t test* were used for testing differences in means of SB.

**Table 10a.**
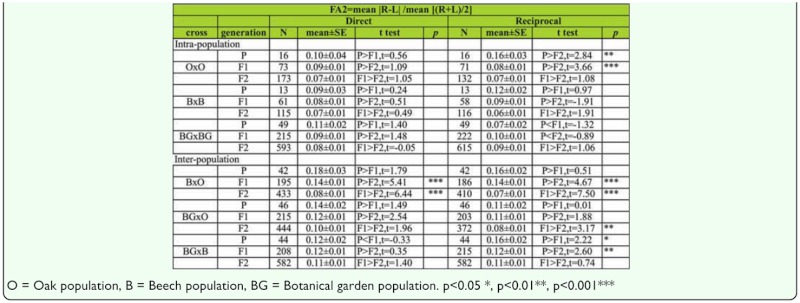
The FA2 index differences between generations and type of crosses for sternopleural bristles in males (direct and reciprocal crosses); *t* *test* were used for testing differences in mean of SB.

**Table 10b.**
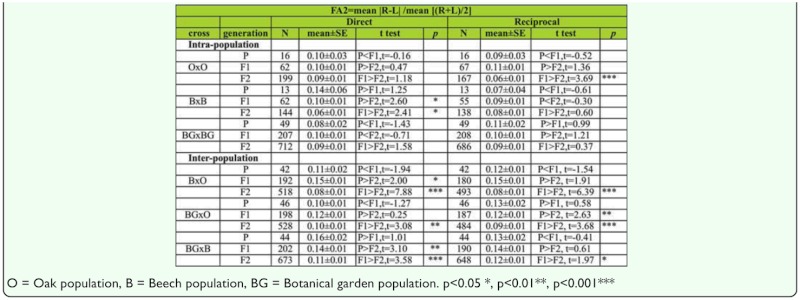
The FA2 index differences between generations and type of crosses for sternopleural bristles in females (direct and reciprocal crosses); *t test* were used for testing differences in means of SB.

## Abbreviations

**DI**,: developmental instability;
**FA**,: fluctuating asymmetry;
**V_p_**,: phenotypic variance
